# Synergistic ferroptosis‐starvation therapy for bladder cancer based on hyaluronic acid modified metal–organic frameworks

**DOI:** 10.1002/btm2.10515

**Published:** 2023-03-22

**Authors:** Yu Wang, Kunfeng Xie, Wei Chen, Yunze Fang, Qixin Mo, Henghui Zhang, Xinlei Zhao, Dongqing Li, Wanlong Tan, Peng Zhao, Fei Li

**Affiliations:** ^1^ Department of Urology Nanfang Hospital, Southern Medical University Guangzhou Guangdong 510515 People's Republic of China; ^2^ Department of Urology Zigong Fourth People's Hospital Zigong Sichuan People's Republic of China; ^3^ Guangdong Provincial Key Laboratory of New Drug Screening, NMPA Key Laboratory for Research and Evaluation of Drug Metabolism, Guangdong Provincial Key Laboratory of Cardiac Function and Microcirculation School of Pharmaceutical Sciences, Southern Medical University Guangzhou 510515 People's Republic of China

**Keywords:** bladder cancer, ferroptosis, hyaluronic acid, metal–organic framework, starvation therapy

## Abstract

Bladder cancer (BCa) is one of the most common malignancies of the urinary tract. Metastasis and recurrence of BCa are the leading causes of poor prognosis, and only a few patients can benefit from current first‐line treatments such as chemotherapy and immunotherapy. It is urgent to develop more effective therapeutic method with low side effects. Here, a cascade nanoreactor, ZIF‐8/PdCuAu/GOx@HA (ZPG@H), is proposed for starvation therapy and ferroptosis of BCa. The ZPG@H nanoreactor was constructed by co‐encapsulation of PdCuAu nanoparticles and glucose oxidase into zeolitic imidazolate framework‐8 (ZIF‐8) modified by hyaluronic acid. The vitro results indicated that ZPG@H enhanced intracellular reactive oxygen species levels and reduced mitochondrial depolarization in the tumor microenvironment. Therefore, the integrated advantages of starvation therapy and chemodynamic therapy endow ZPG@H with a perfect ferroptosis inducing ability. This effectiveness, combined with its excellent biocompatibility and biosafety, means that ZPG@H could make a critical contribution to the development of novel BCa treatments.

**Abbreviations**
BCa: bladder cancer
CDT: chemodynamic therapy
GOx: glucose oxidase
HA: hyaluronic acid
ROS: reactive oxygen species
ZIF‐8: zeolitic imidazolate framework‐8
ZPG@H: ZIF‐8/PdCuAu/GOx@HA

## 1 INTRODUCTION

Bladder cancer (BCa) is one of the most common urinary tract malignancies. According to the National Comprehensive Cancer Network Clinical Practice Guidelines, metastasis and recurrence of BCa are the main causes of poor prognosis, especially in patients who are diagnosed at late stages.^1^, ^2^ In recent years, cisplatin (CDDP)‐based chemotherapy and immune checkpoint inhibitors have been used as first‐line treatments for muscle‐invasive and metastatic BCa; however, it has been estimated that only 50% of BCa patients benefit from chemotherapy and 20% benefit from immunotherapy.^3^ The overall survival of patients with BCa remains unsatisfactory because of adverse effects, resistance, and intolerance.^4^, ^5^ Therefore, novel antitumor treatments that are less toxic and resistant are urgently needed.

Ferroptosis is an iron‐dependent form of programmed cell death characterized by lipid peroxidation, which is usually caused by an imbalance in the generation and degradation of intracellular reactive oxygen species (ROS).^6^, ^7^ The increase in intracellular ROS and decrease in the mitochondrial volume are considered biomarkers of ferroptosis.^8^, ^9^, ^10^ Ferroptosis is reported to be a natural barrier to cancer development because certain tumor suppressors, such as p53 and BRCA 1‐associated protein 1 (BAP1), engage in the ferroptosis pathway.^11^, ^12^ Moreover, the high load of ROS and specific mutations in tumor cells make them intrinsically more susceptible to ferroptosis.^10^ Therefore, ferroptosis is an important target for BCa treatment. Nanomaterials are essential ferroptosis inducers because of their high ROS production efficiency, potential for glutathione (GSH) depletion, and other features.^13^ On the one hand, the specific modifications on the surface of nanomaterials endow them with long blood circulation time and reduced renal clearance. Conversely, nanomaterials with passive and active target abilities can enhance accumulation at the tumor site and reduce adverse effects.^14^ For example, Liao et al. reported a nanodrug that specifically targets BCa cells to deliver iron ions, produce excess ROS, and ultimately lead to ferroptosis.^15^ Moreover, AuNRs, IONs@Gel,^16^ and gold clusters (PAA4 and PAA5)^17^ have also been reported have potential for BCa treatment targeting the ferroptosis pathway. Therefore, it is significant to develop nanomedicine that can actively target BCa and induce subsequent ferroptosis pathway.

Chemodynamic therapy (CDT) has been designed to convert endogenous chemical energy into ROS via Fenton and Fenton‐like reactions,^18^, ^19^ ultimately resulting in cell apoptosis or even ferroptosis.^20^ However, a single CDT treatment usually does not completely eradicate tumors because of low ROS generation efficiency.^21^, ^22^, ^23^, ^24^ Therefore, a CDT combined with a synergistic treatment strategy should be developed. Owing to the Warburg effect, the proliferation of cancer cells consumes more glucose than normal tissues.^25^ Once glucose supply is interrupted, the growth of tumor cells is inhibited.^25^, ^26^ Thus, starvation therapy, which is related to glucose metabolism, is increasingly being regarded as a potential clinical therapy approach.^27^ As glucose oxidase (GOx) can catalyze the oxidation of glucose into gluconic acid and hydrogen peroxide,^28^ it has been reported to be involved in the regulation of tumor metabolism and can play a role in starvation therapy. More importantly, GOx‐based starvation therapy can synergistically enhance the efficiency of CDT by increasing H_2_O_2_ levels in the tumor microenvironment.^29^, ^30^, ^31^ Moreover, Zhou and his colleagues indicated that GOx/BSO@CS PVs effectively inhibit the growth of 4T1 tumors and provide the basis of a promising strategy to prepare pH‐sensitive nanomedicines for synergistic starvation‐ferroptosis tumor therapy.^32^

Here, a cascade nanoreactor intended for starvation therapy and ferroptosis of BCa was proposed. The nanoreactor ZPG@H was constructed by co‐encapsulation of PdCuAu nanoparticles and GOx into zeolitic imidazolate framework‐8 (ZIF‐8) modified with hyaluronic acid (HA). After entering the tumor environment, HA‐modified ZPG@H can specifically target the cluster determinant 44 (CD44) receptor, which is expressed on the surface of BCa cell.^33^, ^34^, ^35^ After HA has been decomposed by intracellular hyaluronidase (Hyase),^36^ cascade reactions occurring as glucose were oxidized by O_2_ with GOx as a catalyst, and the produced H_2_O_2_ was converted to a large amount of ROS to progress ferroptosis therapy. The in vitro and in vivo results indicated that the ZPG@H‐based synergistic treatment could greatly inhibit BCa through metabolism regulation combined with lipid peroxidation and high levels of ROS. The integrated advantages of cascaded reaction of glucose through starvation therapy and CDT would greatly induce the ferroptosis of bladder tumor cells. Moreover, ZPG@H showed satisfactory biosafety, as the surface‐coated HA possessed proven biocompatibility and biodegradability.

## 2 EXPERIMENTAL SECTION

### 2.1 Reagents and materials

All the reagents and chemicals used were at least analytical grade and were commercially available. Deionized water (DI water, 18.2 MΩ cm) obtained from a Milli‐Q water system was used for all the experiments. Chloroauric acid hydrate (HAuCl_4_·4H_2_O), cupric chloride hydrate (CuCl_2_·2H_2_O), palladium chloride (K_2_PdCl_4_), hydrogen peroxide (H_2_O_2_), 3,3,5,5‐tetramethylbenzidine (TMB), zinc nitrate hexahydrate (≥98%, Zn(NO_3_)_2_∙6H_2_O), HA, were obtained from Sigma‐Aldrich Co. Cells counting kit (CCK‐8) was gained from GDSBio (Guangzhou, China). JC‐1 probe was attained from MedChemExpress (Shanghai, China). Dihydroethidium (DHE, ROS probe) was obtained from UElandy Inc. (Suzhou, China). Calcein‐AM and propidium iodide (PI) were acquired from BestBio (Shanghai, China). Liperfluo (lipid peroxidation probe) was obtained from Dojindo (Kumamoto, Japan). 4,6‐diamino‐2‐phenyl indole (DAPI) was obtained from Bioscience (Shanghai, China). Fetal bovine serum (FBS), streptomycin, penicillin, trypsin EDTA solution, Dulbecco's Modified Eagle's Medium (DMEM), high‐glucose medium, and PBS were purchased from Gibco (Gibco Company, USA). SLC7A11 rabbit polyclonal antibody, SLC3A2 mouse polyclonal antibody, GPX4 mouse monoclonal antibody, GAPDH rabbit monoclonal antibody, HRP‐conjugated goat anti rabbit IgG (H + L) antibody, and HRP‐conjugated goat anti mouse IgG (H + L) antibody were acquired from Proteintech (Wuhan, China). Deionized water (DI water, 18.2 MΩ cm) was obtained from the Milli‐Q water system for all the experiments.

Instruments: UV–Vis absorbance spectrum was recorded on a UV‐5500PC UV–Vis spectrophotometer (Shanghai METASH, China). Transmission electron microscope (TEM) images were obtained on a JEM‐2100F TEM (JEOL, JEM‐2100F, Japan) under 200 kV. The flow cytometric analysis was performed by a flow cytometry (BD FACSCanto II, USA). Fluorescence inverted microscope (FIM) images were obtained on an FIM (Axio Observer, Germany). The cell viability and hemolysis assay were measured using multifunctional microplate reader (Infinite M1000 Pro, Tecan, Switzerland). Fourier transform infrared (FT‐IR) spectroscopy was recorded with a Tensor 27 FT‐IR spectrometer (Bruke, Germany).

### 2.2 Ethics statement

All animal studies were carried out in strict accordance with the Guide for the Care and Use of Laboratory Animals as adopted by the Institutional Animal Care and Use Committee of Southern Medical University.

### 2.3 Cell culture

Human BC cell lines T24 were used in this study. The cells were cultured in DMEM medium (Gibco, USA) supplemented with 10% FBS (Gibco, USA) and incubated at 37°C in 5% CO_2_. The cell lines were authenticated by short tandem repeat (STR) profiling before receipt and were propagated for 6 months after resuscitation.

### 2.4 Synthesis of ZIF‐8/PdCuAu/GOx@HA

The preparation of PdCuAu nanoparticles were synthesized as follows. K_2_PdCl_4_, CuCl_2_, and HAuCl_4_ aqueous solution with the molar ratio of 1:1:1 was added to 9.1 mL of the mixed solution containing 0.256 g CTAB and 0.21 g citric acid. The mixed solution was stirred for several minutes, then 0.03 g ascorbic acid was added and stirred for 20 min. It was then transferred to stainless steel autoclave lined with polytetrafluoroethylene. The sealed vessel was heated at 190°C for 4 h and then cooled to room temperature. The product was separated by 9000 rpm for 10 min and washed with deionized ethanol treated three times.

Then, 3 mg PdCuAu nanoparticles and 5 mg GOx were dispersed in 1 mL Milli‐Q water, which were mixed with 15 mL Zn (NO_3_)_2_ (30 mM) and 2‐methylimidazole (100 mM). After stirring for 30 min, the primary product ZIF‐8/PdCuAu/GOx was centrifuged at a centrifugal rate of 8000 rpm for 10 min and purified by rinsing/centrifugation cycles with water for three times. Finally, 1 mg/mL of HA was reacted with 1 mg/mL ZIF‐8/PdCuAu/GOx, the resultant ZIF‐8/PdCuAu/GOx@HA (ZPG@H) were gained and collected.

### 2.5 Cellular uptake ability of ZIF‐8/PdCuAu/GOx@HA

T24 cells were seeded in confocal dish at a density of 5 × 10^3^ cells and cultured for 24 h. After the T24 cells incubated with RhB‐conjugated ZPG@H for 0, 2, 4, 6, 8, and 10 h, the cells were fixed with 4% paraformaldehyde, sequentially stained with DAPI, and finally observed with a CLSM. Additionally, the treated cells were collected and analyzed with flow cytometry assay (BD FASCVerse, USA).

### 2.6 In vitro cytotoxicity studies

In order to detect the cell cytotoxicity of ZIF‐8/GOx@HA (ZG@H), ZPG@H, or ZIF‐8/PdCuAu@HA (ZP@H) for T24 cells, we first seeded T24 cells in 96‐well plates. After 24 h, the DMEM containing ZG@H, ZPG@H, or ZP@H at different concentrations (0, 20, 40, 60, 80, 100, and 120 μM) was added to each well and co‐incubated for 24 h. After that, cell viability was quantified with CCK8 assay through a microplate reader.

Additionally, in order to detect PTT of ZPG@H for T24 cells, the cells were planted in 96‐well plates, and the non‐NIR groups were treated with ZPG@H NPs as described above. As for NIR irradiation groups, the cells were pre‐incubated with these NPs for 6 h, and then irradiated with an NIR laser (1064 nm, 1 W/cm^2^, 10 min), and finally incubated for another 18 h. Afterwards, cell viability was quantified with CCK8 assay through a microplate reader.

For the live/dead staining assay, the T24 cells were seeded in 6‐well plates and cultured overnight. Subsequently, different preparations of ZG@H, ZPG@H, or ZP@H were added and incubated for 12 h. After that, the treated cells were stained with Calcein‐AM/PI double stained kit, and finally observed via fluorescence microscopy and evaluated through flow cytometry.

### 2.7 Detection of mitochondrial function

The mitochondrial transmembrane potential was detected with JC‐1 mitochondrial membrane potential (MMP) Assay Kit. Briefly, after treated with ZG@H, ZPG@H or ZP@H, with or without NIR laser, the T24 cells were collected and incubated with JC‐1 staining. Then, the cell samples were observed and imaged with fluorescence microscope, as well as analyzed through flow cytometry assay.

### 2.8 Detection of ROS

The reactive oxygen species was detected with DHE (ROS probe). T24 cells treated with ZG@H, ZPG@H, or ZP@H, with or without NIR laser, were harvested and incubated with DHE. After that, the cells were observed via fluorescence microscope and assessed by flow cytometry assay.

### 2.9 Detection of the level of lipid peroxidation

In order to further evaluate the level of lipid peroxidation, the treated cells were collected and detected with via liperfluo probe, and finally observed with fluorescence microscope and measured by flow cytometry.

### 2.10 Western blotting

Protein lysates were separated using 10% SDS‐PAGE and transferred onto nylidene difluoride, a PVDF membrane (Roche, Switzerland). Then, membranes were incubated with antibodies against SLC7A11, SLC3A2, GPX4, and GAPDH (1:1000, Proteintech, China), followed by incubation with secondary antibodies. Finally, the proteins were detected using an enhanced chemiluminescence (ECL, Pierce, Rockford, IL, USA) detection system.

### 2.11 In vivo therapeutic effect of ZPG@H

Three groups of the BCa‐bearing mice mentioned above were also used to evaluate the therapeutic effect of ZPG@H. The tumor‐bearing mice were injected with PBS, 10 or 20 mg/kg ZPG@H via tail vein on days 7, 14, 21, and 28, and the laser‐treated groups were performed by a 1064 nm laser after 3 h post‐injection. Tumor size and mouse weight were recorded every 4 days. Tumor volume was recorded from the first day of injection until the end of the experiment and calculated as follows: length × width^2^ × 0.5. On day 35, the mice were sacrificed and the tumors were harvested for imaging. Additionally, the tumor tissues were harvested, and fixed in 4% formalin for H&E (Solaibao, China), SLC7A11 (Proteintech, China) staining.

### 2.12 In vivo biocompatibility of ZPG@H

Twenty female BALB/c mice were prepared to evaluate the biocompatibility of ZPG@H. The mice were randomly divided into four groups: (1) PBS, (2) injected 20 mg/kg NPs1 day before death, (3) injected 20 mg/kg NPs 7 days before death, (4) injected 20 mg/kg NPs 14 days before death. The blood samples of mice in all groups were collected and performed relevant biochemical index assay, which was consigned to Servicebio (Wuhan, China).

Additionally, the hearts, livers, spleens, lungs, and kidneys of all groups BCa‐bearing mice mentioned above were collected and fixed in 4% formalin for H&E to assess the biocompatibility.

### 2.13 Statistical analysis

All data are expressed as the mean ± standard deviation (*n* ≥ 3), and the significance of differences among groups was evaluated using one‐way ANOVA and a Student's *t*‐test (*p < 0.05, **p < 0.01, ***p < 0.001).

## 3 RESULTS

### 3.1 Characterization and ROS production of ZIF‐8/PdCuAu/GOx@HA (ZPG@H)

**FIGURE 1:**
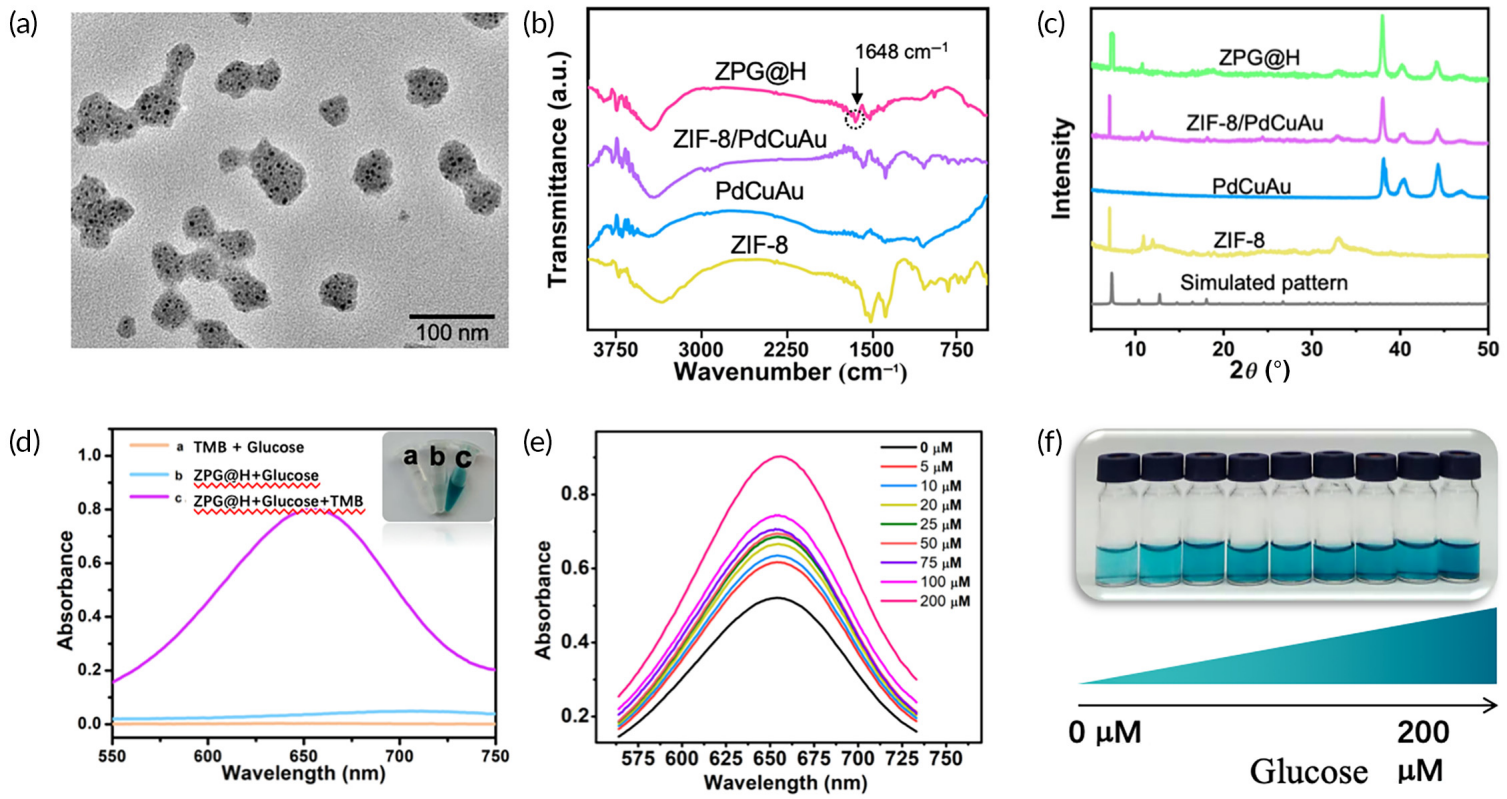
The transmission electron microscope image of ZPG@H. (a) The Fourier transform infrared spectra (b) and X‐ray diffraction results (c) of ZIF‐8, PdCuAu, ZIF‐8/PdCuAu, and ZPG@H. The UV–Vis spectra of glucose with different additions in the PBS (0.1 M, pH = 6.5) (d). The UV–Vis spectra of TMB and ZPG@H with different concentrations of glucose from 0 to 200 M (e). (f) The corresponding photograph of figure (e).

The morphology and structure of ZPG@H were characterized using TEM, FT‐IR, and X‐ray diffraction (XRD). The TEM images showed that the obtained ZPG@H had an average size of approximately 85 nm (Figure 1a), and PdCuAu nanoparticles with a diameter of 5 nm were uniformly distributed inside the ZIF‐8 framework. FT‐IR spectroscopy was used to analyze the functional groups of the ZPG@H nanocomposites (Figure 1b); 1567 cm^−1^ showed the vibration absorption of the imidazole framework, 3450 cm^−1^ showed the vibration absorption peak of —OH in water molecules, and 1549 cm^−1^, the vibration absorption peak of the C=C double bond on the benzene ring,^37^ indicating that the ligand was completely deprotonated at this position and providing the necessary conditions for the preparation of the ZIF‐8 crystal.^38^ In addition, the peak at 1648 cm^−1^ can only be observed in the IR spectrum of ZPG@H, and this was corresponding to the N—H and C—H stretching of GOx,^39^ confirming the successful preparation of ZPG@H. The XRD patterns of pure ZIF‐8, PdCuAu, ZIF‐8/PdCuAu, and ZPG@H are shown in Figure 1c. In addition to the decrease in diffraction intensity, there is no obvious displacement change in the diffraction angle, so there is no evident change in the ZIF‐8 crystal, which proves that the encapsulation of PdCuAu and GOx does not destroy the crystal structure of ZIF‐8. All the results confirmed that the experimental materials had been successfully prepared.

To verify the ROS production capability of ZPG@H in the presence of glucose, the typical substrate TMB was used as a chromogenic substrate. As shown in Figure 1d, in the presence of glucose, ZPG@H effectively catalyzed the oxidation of colorless TMB to blue ox‐TMB (curve c). However, in the control group, without the addition of ZPG@H (curve a) or glucose (curve b), there was no obvious color change or absorbance at 652 nm, indicating that both ZPG@H and glucose are required for the reaction. The UV–Vis spectra of TMB and ZPG@H after reacting with different glucose concentrations for 30 min were recorded. As shown in Figure 1e, the absorbance intensity at 652 nm gradually increased with increasing glucose concentration, and the blue color of the solution deepened (Figure 1f). As TMB is easily oxidized by hydroxyl radicals (•OH), it can be estimated that the increased absorbance intensity at 652 nm results from the cascade catalytic reaction of ZPG@H. In this process, glucose was quickly oxidized to H_2_O_2_ in the presence of GOx, and H_2_O_2_ was converted to •OH by PdCuAu through a Fenton‐like reaction. The above results show that ZPG@H has excellent potential to induce starvation and ROS‐related ferroptosis in the tumor microenvironment. The Michaelis–Menten constants (*K*_m_), an important indicator of catalytic activity, was determined and calculated for the free GOx and the encapsulated GOx. Figure S1 shows the double inverse relationships between the glucose concentration and initial reaction rate for two formations. Then, the *K*_m_ value can be calculated as 8.23 mM for free GOx and 9.24 mM for encapsulated GOx, based on the equation *K*_m_ = Slope/Intercept.^40^ The above result implied that the catalytic activity of GOx slightly decreased after the formation of ZPG@H, but there was not a significant difference between the two formations.

The ratio of GOx and PdCuAu nanoparticles was optimized; 3 mg PdCuAu nanoparticles and GOx of different masses (3, 4, 5, or 6 mg) were used for the synthesis of ZPG@H. Figure S2 reveals that absorbance intensity increased with the percentage of GOx increase and reached to a platform at 5 mg. Therefore, it was thought that 3 mg GOx and 5 mg PdCuAu nanoparticles were the optimal ratio to synthesize ZPG@H.

DLS measurements of ZPG@H in different mediums or in PBS with different pH values were conducted. As shown in Figure S3A, ZPG@H dispersed well in DI water, PBS (pH 7.4), DMEM, and 10% serum for at least 12 h. The hydrodynamic diameters of ZPG@H hardly changed after incubation for 12 h in different mediums (Figure S3B). During the study of the stability of ZPG@H in PBS with different pH values, Figure S3C,D reveal that the hydrodynamic particle size of ZPG@H is ultra‐stable at pH = 7.5 and pH = 6.5, just slightly increased after incubation for 12 h in acidic solution (pH = 5.5). The above results indicated the satisfying application of ZPG@H in physiological environments and further proved the stability of ZPG@H in various solutions.

### 3.2 In vitro therapeutic efficacy and cellular internalization of ZIF‐8(PdCuAuGOx)@HA (ZPG@H)

**FIGURE 2:**
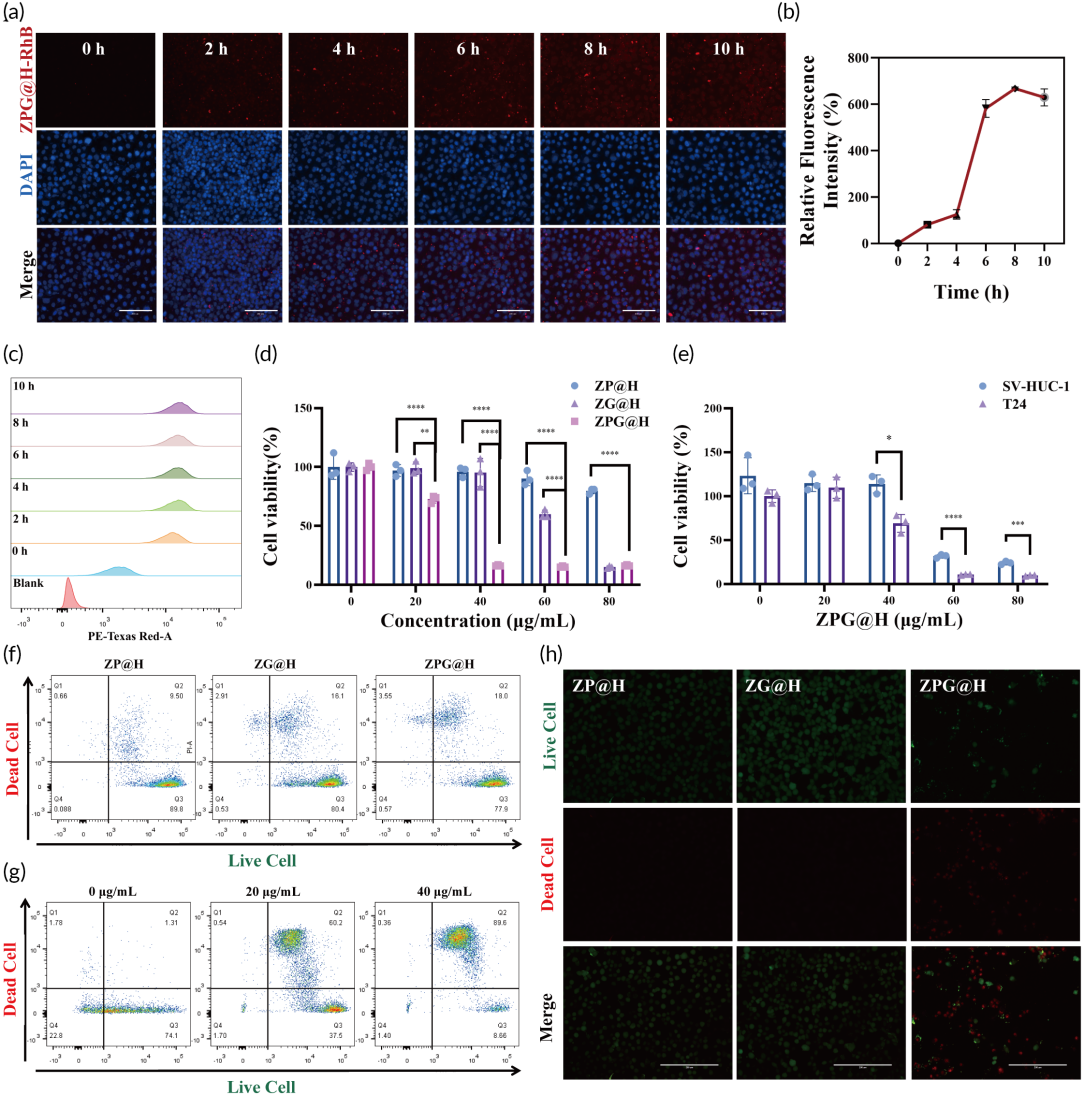
In vitro assessments of cell death induced by ZPG@H. (a) Fluorescence imaging of RhB‐conjugated tumor cells after exposure to ZPG@H (30 μg/mL) for 10 h. All scale bars are 200 μm. (b) Average PE‐Texas Red fluorescence intensities of tumor cells treated with 30 μg/mL of ZPG@H for 10 h. (c) Flow cytometry analysis of RhB‐conjugated tumor cell after treated with ZPG@HA for 10 h. (d) Cell viability after treatments with different concentrations of ZG@H, ZP@H and ZPG@H for 24 h. (e) Cell viability of T24 and SV‐HUC‐1 cells after treatments with different concentrations of ZPG@H for 12 h. (f) Fluorescence imaging of T24 cells treated with ZG@H, ZP@H, and ZPG@H (30 μg/mL) for 6 h. Live/dead stain showing dead cells as red and live cells as green. The scale bar is 200 μm. (g) Flow cytometry analysis of cell viability after treated with ZG@H, ZP@H, and ZPG@H (30 μg/mL) for 6 h. (h) Flow cytometry analysis of cell viability after treated with different concentrations of ZPG@H for 12 h.

The CD44 receptor is typically highly expressed on the surface of BCa cells (e.g., T24); however, it is barely expressed in normal cell lines (e.g., SV‐HUC‐1 cells). Moreover, HA‐conjugated NPs preferentially targeted CD44 receptor‐overexpressing cells. We explored the cellular uptake of ZPG@H labeled with rhodamine B (RhB) to investigate its active targeting ability through CD44 receptor‐mediated endocytosis. As shown in Figure 2a,b, the red fluorescence gradually increased as the incubation time increased from 0 to 10 h. These results indicate that our nanoplatform may be specifically internalized by T24 cells through overexpression of CD44 receptors. Moreover, the red fluorescence intensity in T24 cells treated with RhB for different incubation times was estimated using flow cytometry. Figures 2c and S4A show that RhB‐labeled ZPG@H‐treated T24 cells exhibited a high uptake rate with time‐dependent properties. These results confirmed the efficient internalization of ZPG@H and guaranteed the cascade of catalytic reactions inside the T24 cell.

Next, T24 cells treated with three groups of ZG@H, ZP@H, and ZPG@H were used to analyze the antitumor effect in vitro via the CCK8 assay (Figure 2d). Dose‐dependent viability was observed in T24 cells incubated with different drug formulations. As a result of starvation therapy, cell viability was reduced to 95% by 30 μg/mL in the ZG@H group. Additionally, the viability of T24 cells decreased to 96% at 40 μg/mL when cultivated with ZP@H for 24 h. Moreover, remarkable cytotoxicity was observed in the synergistic therapy group ZPG@H compared with the monotherapy groups. The IC_50_ of the three kinds of nanomedicine were 63.66, 109.00, and 26.88 μg/mL, respectively. In contrast, SV‐HUC‐1 cells showed a higher tolerance toward 40 μg/mL ZPG@H (Figure 2e). These results suggest that ZPG@H has excellent biosafety toward normal cells and has the potential to treat CD44 overexpressed T24 cells.

The cytotoxicities of ZG@H, ZP@H, and ZPG@H were measured using the Calcein‐AM and PI staining methods. As shown by inverted fluorescence microscopy (Figures 2f and S4E), T24 cells in the ZPG@H group exhibited the highest level of mortality. Moreover, flow cytometry revealed that T24 cells treated with ZPH@H showed a higher percentage of apoptosis than the other groups (Figures 2g and S4F). Together, these results demonstrate the excellent killing effect of ZPG@H on T24 cells in vitro. In addition, the flow cytometry results, which were consistent with fluorescence microscopy, revealed that ZPG@H had the best therapeutic efficacy among the three drug formulations, and its cytotoxicity was enhanced with increasing concentration (Figures 2h and S4B–D).

### 3.3 In vitro ROS production and ZPG@H starvation therapy

**FIGURE 3:**
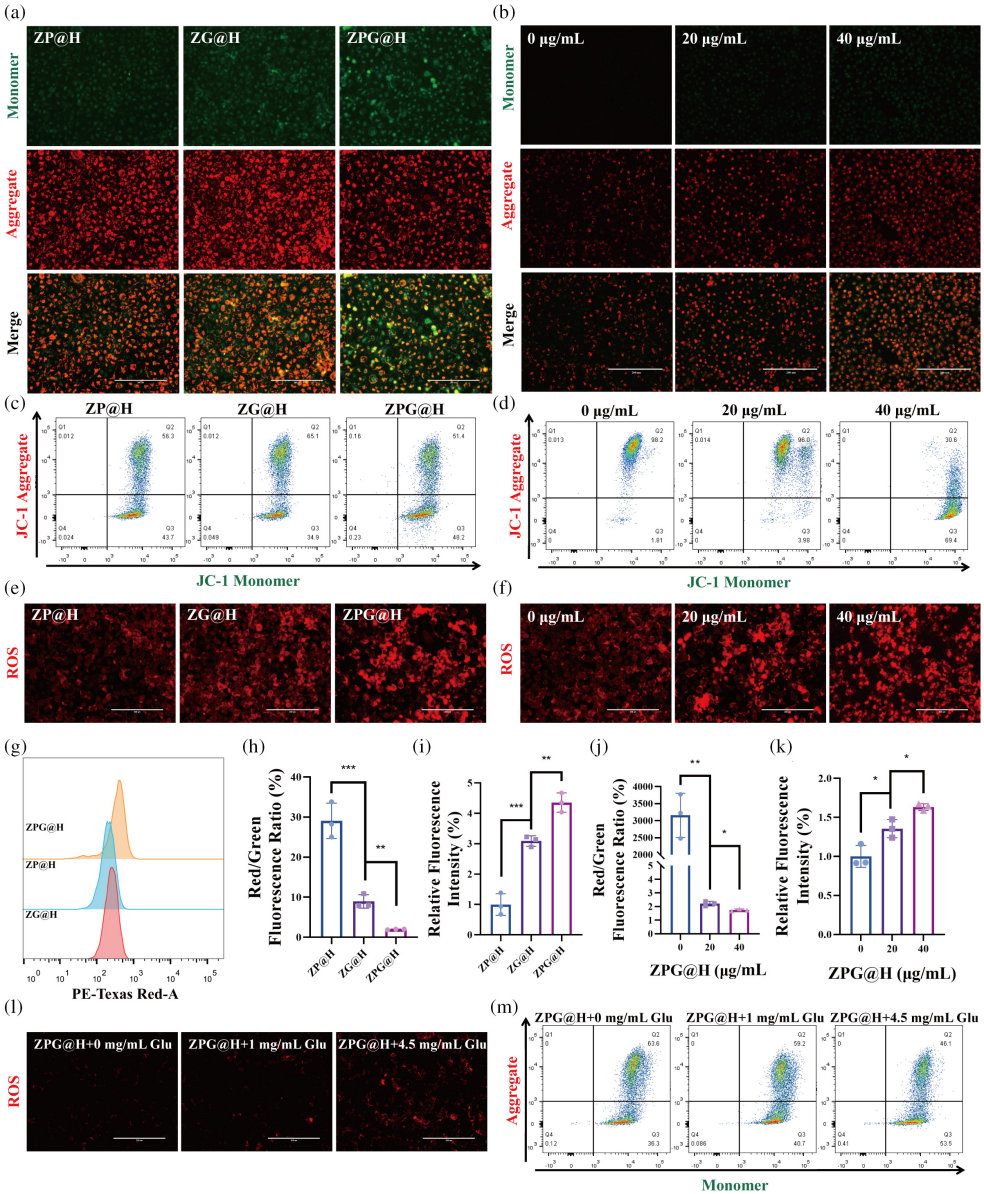
Cell death mechanism caused by ZPG@H. (a) Fluorescence imaging of JC‐1 stained T24 cells treated with ZG@H, ZP@H and ZPG@H (30 μg/mL) for 6 h. The scale bar represents 200 μm. (b) Fluorescence imaging of JC‐1 stained T24 cells incubated with different concentrations of ZPG@H for 6 h. The scale bar represents 200 μm. (c) Flow cytometry analysis of JC‐1 stained T24 cells treated with ZG@H, ZP@H, and ZPG@H (30 μg/mL) for 6 h. (d) Flow cytometry analysis of JC‐1 stained T24 cells incubated with different concentrations of ZPG@H for 6 h. (e) Fluorescence imaging of reactive oxygen species (ROS)‐probe‐stained T24 cells treated with ZG@H, ZP@H, and ZPG@H (30 μg/mL) for 6 h. The scale bar represents 200 μm. (f) Fluorescence imaging of ROS‐probe‐stained T24 cells incubated with different concentrations of ZPG@H for 6 h. The scale bar represents 200 μm. (g) Flow cytometry analysis of ROS‐probe‐stained T24 cells treated with ZG@H, ZP@H, and ZPG@H (30 μg/mL) for 6 h. (h) Average red/green fluorescence ratio of JC‐1 stained T24 cells treated with ZG@H, ZP@H, and ZPG@H (30 μg/mL) for 6 h. (i) Average PE‐Texas Red fluorescence intensities of ROS‐probe‐stained T24 cells treated with ZG@H, ZP@H, and ZPG@H (30 μg/mL) for 6 h. (j) Average red/green fluorescence ratio of JC‐1 stained T24 cells incubated with different concentrations of ZPG@H for 6 h. (k) Average PE‐Texas Red fluorescence intensities of ROS‐probe‐stained T24 cells incubated with different concentrations of ZPG@H for 6 h. (l) Fluorescence imaging of ROS‐probe‐stained T24 cells treated with different concentration glucose and ZPG@H (30 μg/mL) for 6 h. The scale bar represents 200 μm. (m) Flow cytometry analysis of JC‐1 stained T24 cells after exposure to different concentration glucose and ZPG@H (30 μg/mL) for 6 h. Asterisks indicate significant differences (*p < 0. 05; **p < 0.01; ***p < 0.001).

Previous studies have reported that ROS can cause irreversible damage to mitochondria.^41^ To further assess the influence of ROS on the mitochondria of T24 cells, a crucial indicator of mitochondrial function, the MMP, was detected using the JC‐1 fluorescent probes, respectively. The fluorescence of JC‐1 changed from red to green when MMP decreased. Furthermore, aggregated JC‐1 appears as red fluorescence under intact membrane potential, whereas dispersed JC‐1 appears as green fluorescence under disrupted membrane potential.^42^ As a result, decreased JC‐1 red fluorescence was observed in T24 cells incubated with those three drug formulations, especially with the synergistic therapy group of ZPG@H (Figure 3a,h). The same result was obtained using flow cytometry (Figures 3c and S5A). Moreover, concentration‐dependent MMP damage was also exhibited in the fluorescent imaging (Figure 3b,j) and flow cytometry (Figures 3d and S5B) results of JC‐1. The DHC probe was used to evaluate ROS levels in T24 cells in the different groups. Figure 3e,g shows that the lowest ROS level was observed in the ZP@H group, while the starvation therapy group had a higher ROS level. Notably, the highest red fluorescence intensity was observed in the T24 cells after incubation with ZPG@H (Figure 3i). In addition, after incubation with different concentrations of ZPG@H, the red fluorescence increased as the concentration of ZPG@H increased from 0 to 40 g/mL (Figure 3f,k). However, flow cytometry detected a sharp decline in ROS at a concentration of 40 μg/mL, which was probably caused by numerous cell deaths (Figure S5C).

To evaluate starvation treatment, T24 cells were incubated with 30 g/mL ZPG@H in a medium containing different glucose concentrations. The highest ROS level was detected in T24 cells cultivated in a medium with 4.5 mg/mL glucose, whereas weak red fluorescence was observed in cells cultivated in a medium without glucose (Figure 3l). Similarly, reduced red fluorescence of JC‐1 was observed in T24 cells incubated with increasing glucose concentration in the culture medium (Figure 3m). The above results confirm that ZPG@H can produce large amounts of ROS through the cascaded catalytic reaction of the CDT effect of PdCuAu nanoparticles and GOx starvation therapy.

### 3.4 Ferroptosis of BCa cells based on ZPG@H

**FIGURE 4:**
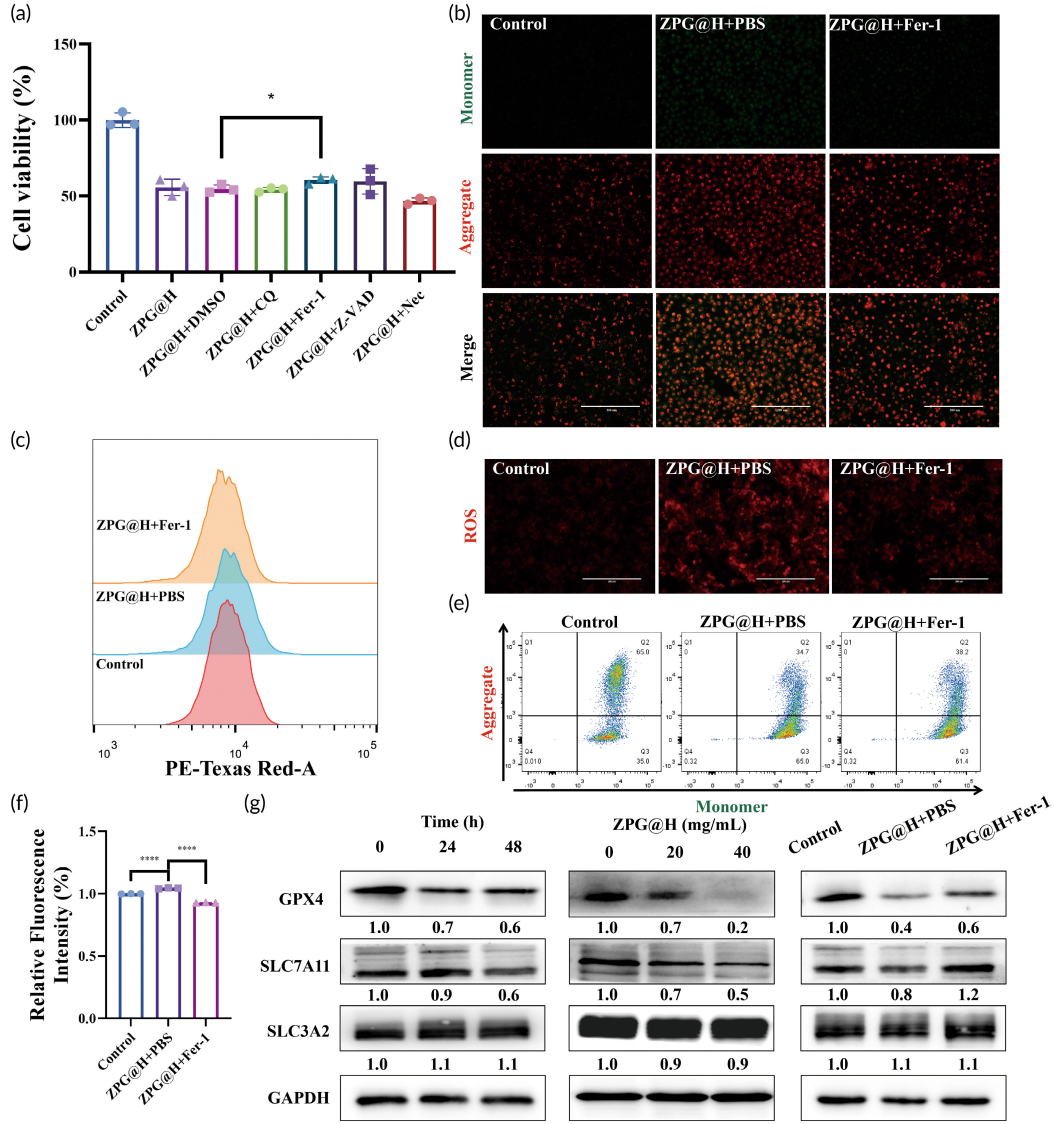
ZPG@H caused cell death via ferroptosis. (a) Cell viabilities of T24 cells treated with ZPG@H (30 μg/mL) and different inhibitors (cell viabilities of cells only treated with PBS was set as 100%). (b) Fluorescence imaging of JC‐1 stained T24 cells treated with ZPG@H (30 μg/mL) and Fer‐1 (20 μM) for 6 h. The scale bar represents 200 μm. (c) Flow cytometry analysis of reactive oxygen species (ROS)‐probe‐stained T24 cells incubated with ZPG@H (30 μg/mL) and Fer‐1 (20 μM) for 6 h. (d) Fluorescence imaging of ROS‐probe‐stained T24 cells incubated with ZPG@H (30 μg/mL) and Fer‐1 (20 μM) for 6 h. (e) Flow cytometry analysis of JC‐1 stained T24 cells treated with ZPG@H (30 μg/mL) and Fer‐1 (20 μM) for 6 h. (f) Average PE‐Texas Red fluorescence intensities of ROS‐probe‐stained T24 cells incubated with ZPG@H (30 μg/mL) and Fer‐1 (20 μM) for 6 h. Asterisks indicate significant differences (*p < 0. 05; **p < 0.01; ***p < 0.001). (g) Protein expression levels of SLC7A11, GPX4, and SLC3A2 after treated with different concentrations of ZPG@H for 24 h, 30 μg/mL ZPG@H for different time and with or without Fer‐1 (20 μM). GAPDH was used as an internal control.

To provide further evidence on the death mechanism of T24 cells induced by ZPG@H, a CCK8 assay was conducted to evaluate T24 cell viability in the presence of various inhibitors, including autophagy inhibitors chloroquine (CQ), ferroptosis inhibitors (Ferrostatin‐1, Fer‐1), apoptosis inhibitors (ZVAD‐FMK, ZVAD), and necrosis inhibitor (Necrostatin1, Nec1). As shown in Figure 4a, ZPG@H‐induced cell death was markedly reduced by Fer‐1, implying that ZPG@H induced ferroptosis in T24 cells. To confirm the role of the ferroptosis pathway in ZPG@H‐induced cell death, ROS and JC‐1 probes were used to assess the process of ferroptosis via fluorescence microscopy and flow cytometry. The highest red fluorescence was observed in the group treated with ZPG@H without inhibitor, and the lowest red fluorescence in the group treated with Fer‐1 (Figures 4c,d, and S6A). Meanwhile, the aggregate/monomer fluorescence ratio of the group with inhibitor was higher than that of the group without inhibitor in the fluorescence microscopy and flow cytometry assay results (Figures 4b,e,f, and S6B).

Additionally, LiperFluo was used to evaluate intracellular lipid hydroperoxides. As shown in Figure S6C,E, the green fluorescence of T24 cells incubated with 40 μg/mL ZPG@H was significantly stronger that the groups with 0 and 20 μg/mL ZPG@H. In addition, the increase in lipid hydroperoxide following ZPG@H treatment showed a concentration‐dependence in cytometry assay (Figure S6G,I). In the rescue experiment, ZPG@H‐induced increase of lipid peroxidation was rescued by the iron death inhibitor Fer‐1 both in fluorescence microscopy and cytometry assay (Figure S6D,F,H,J).

Western blotting was performed to measure the protein levels of the ferroptosis‐related genes. Interestingly, the protein expression levels of the cystine/glutamate antiporter (SLC7A11) and glutathione peroxidase 4 (GPX4) decreased in T24 cells after ZPG@H treatment. In addition, when T24 cells were co‐cultivated with different concentrations of ZPG@H, the expression of SLC7A11 and GPX4 was reduced as the ZPG@H concentration increased, while SLC3A2 expression remained stable. Moreover, the reduction in the expression levels of these proteins was enhanced by Fer‐1. (Figure 4g). It made certain that the ZPG@H can induce ferroptosis of T24 cells by inhibiting the expression of SLC7A11 protein.

### 3.5 In vivo antitumor effect and enhanced ferroptosis of ZPG@H

**FIGURE 5:**
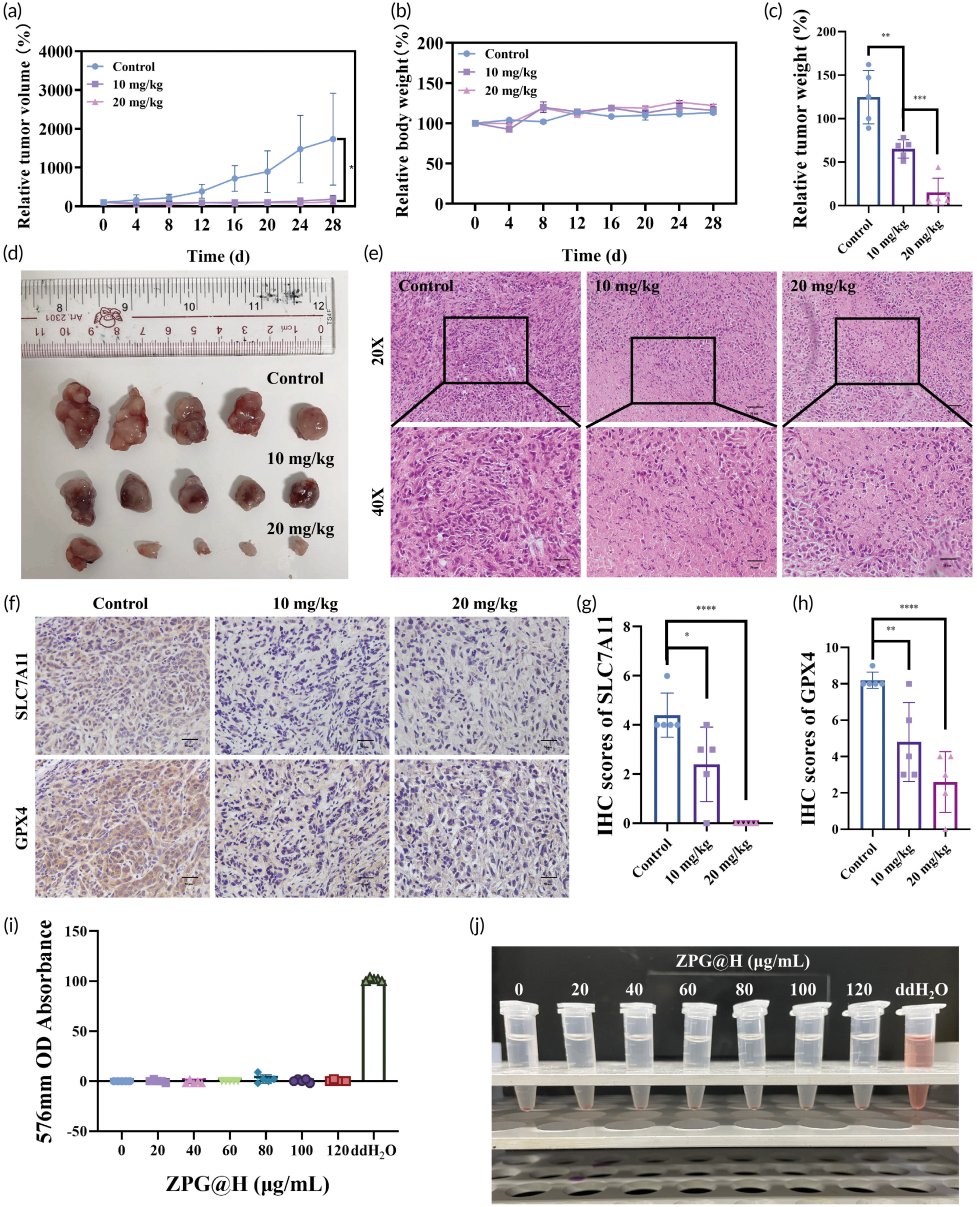
In vivo therapy effect of ZPG@H. (a) Time‐dependent tumor volume changes after different treatments. (b) Weight change curve of mice with time for different treatment groups. (c) Relative weight of tumors in different treatment groups after intervention. (d) Photos of the tumors extracted from mice in different groups at the end of treatments. (e) H&E staining of tumor section in different groups at the end of treatments. (f) IHC staining of tumor section in different groups at the end of treatments. (g) Average IHC scores of SLC7A11 in different groups. (h) Average IHC scores of GPX4 in different groups. (i) Hemolysis rate of red blood cells treated with different concentrations (0, 20, 40, 60, 80, 100, and 120 μg/mL) of ZPG@H. (j) Photographs of system after the reaction with different concentrations of ZPG@H. Asterisks indicate significant differences (*p < 0. 05; **p < 0.01; ***p < 0.001).

Subsequently, the in vivo therapeutic effect was assessed in a T24 tumor‐bearing mouse model. Fifteen T24 tumor‐bearing mice (tumor volume of approximately 100 mm^3^) were randomly divided into three groups: PBS, 10, and 20 mg/kg ZPG@H, and the corresponding agents were injected intravenously into mice (Figure S7A). During the entire treatment period, the weight of mice and the relative tumor volumes were measured and calculated every 3 days. As indicated in Figure 5a,d, compared with the control group, the tumor volumes of the groups treated with ZPG@H were remarkably smaller, and the inhibition rates of the treatment groups were 89.4% and 93% at doses of 10 and 20 mg/kg, respectively. Similarly, the weight of tumor tissues from the high‐dose group was the lowest among the three groups (Figure 5c). Hematoxylin and eosin (H&E) staining of tumors was conducted on different treatment groups, and the results indicated that ZPG@H increased the nuclear fragmentation of cells, indicating a significant killing effect on BCa tissues (Figure 5e). In addition, our results showed that the ZPG@H group had the slowest growth in the three drug formulations (Figure S7B,C). Additionally, compared with control group, the average tumor weigh of ZPG@H group was only 23.06% (Figure S7D). Notably, body weight of mice did not differ significantly between groups (Figure S7E). All results mentioned above revealed that ZPG@H had the best therapeutic effect in those three drug formulations.

After verifying the therapeutic effect of ZPG@H in vivo, the expression of GPX4 and SLC7A11 in tumor tissues suffering from ferroptosis was examined using IHC staining. As shown in Figure 5e,g, the IHC scores of SLC7A11 decreased from 4.4 to 2.4 and 0 for the control, 10 and 20 mg/kg groups, respectively. This revealed that the import of cystine, used in glutathione biosynthesis, decreased in T24 cells treated with ZPG@H. For GPX4, the expression decreased by approximately 58.5% and 31.7% after injection of 10 and 20 mg/kg ZPG@H, respectively (Figure 5f,h). The inhibition of GPX4 can result in lipid peroxidation and ferroptosis. Simultaneously, malondialdehyde levels were elevated in the treated groups, suggesting an important role for the ferroptosis pathway in ZPG@H‐mediated antitumor therapy (Figure S7F). The results showed a marked decrease in SLC7A11 and GPX4, triggered by starvation and ferroptosis in vivo.

Fluorescence imaging was used to detect the biodistribution of Cy5.5 modified ZPG@H in vivo, as shown in Figure S8A,B; ZPG@H can access the tumor region in 3 h. In Figure S8C, the fluorescence can mainly be observed in the tumor, liver, and kidney, revealing that ZPG@H was metabolized through liver and kidney. The metabolism of ZPG@H in the blood of mice was examined by ICP, the Zn^2+^ in blood decreased gradually with time prolongation (Figure S8D), and the decreased trend was consistent with the fluorescence results. *t*_1/2_ was calculated as 12.1 h.

It is vital to evaluate the biosafety and biocompatibility of ZPG@H; therefore, these two qualities must be assessed. Figure 2e shows that the effect of ZPG@H on tumor cells (T24) was more pronounced than that on normal urothelial epithelial cells (SV‐HUC‐1), indicating that the cytotoxicity induced by ZPG@H was selective. In addition, blood compatibility was evaluated using red blood cell (RBCs) hemolytic analysis with mouse RBC. According to our data, hemolysis could hardly be observed with different concentrations of ZPG@H, even at 120 μg/mL. Phosphate‐buffered saline and purified water were used as negative and positive controls, respectively, and the maximum hemolysis rate was less than 5% (Figure 5i,j). In addition, H&E staining of the heart, liver, spleen, lung, and kidney of all groups after the intervention showed little off‐target damage and inflammatory reaction (Figure S9A). Moreover, as shown in Figure S8A, intravenously administered ZPG@H was gradually cleared from the blood over 3 days. Therefore, it is safely eliminated from the body and does not interfere with metal metabolism in the host animals.

As shown in Figure 5b, the weight of the mice increased smoothly during the entire experimental cycle, and there was no significant difference between the group injected with PBS and the groups injected with ZPG@H. In addition, routine blood examination and biochemical analysis demonstrated that the injection had no apparent side effects in the experimental groups (Figure S9B,C). Therefore, based on the above results, it can be deduced that ZPG@H has no obvious side effects in vivo. Taken together, these results confirm the biocompatibility and biosafety of ZPG@H.

## 4 CONCLUSION

In conclusion, the ZPG@H nanocomposite was devised, and its cytotoxicity was produced by promoting starvation therapy and ferroptosis in BCa cells. The cascade reaction of glucose catalyzed by GOx and PdCuAu can produce a large amount of •OH, causing a decrease in MMP and expression of ferroptosis‐related proteins. According to the inhibitor treatment results, cell death caused by ZPG@H occurred mainly via the ferroptosis pathway. An intracellular cystine transporter, SLC7A11, was found to be a potential target of ZPG@H. In addition, when T24 cells were incubated with a certain concentration of ZPG@H, the protein level of SLC7A11 decreased, leading to cytotoxicity through ferroptosis. Altogether, this study provides a synergistic strategy to enhance intracellular ROS levels and reduce mitochondrial depolarization, which has significant value for updating traditional BCa treatments.

## AUTHOR CONTRIBUTIONS

Yu Wang and Kunfeng Xie conceived of the study. Wanlong Tan, Peng Zhao, and Yunze Fang wrote the manuscript. Yu Wang and Wei Chen contributed to the data acquisition and interpretation process. Qixin Mo, Henghui Zhang, and Xinlei Zhao assisted in improving language quality. Fei Li, Yunze Fang, and Wanlong Tan have completed the final revision of the manuscript. All authors have read and approved the final manuscript.

## FUNDING INFORMATION

This study was supported by the Natural Science Foundation of Guangdong Province of China (2021A1515010762, 2023A1515010236) (Fei Li), Outstanding Youth Development Scheme of Nanfang Hospital, Southern Medical University (2019J009) (Fei Li), the President Foundation of Nanfang Hospital, Southern Medical University (2020Z005) (Fei Li), and Beijing Bethune Charitable Foundation (mnzl202017) (Fei Li).

## CONFLICT OF INTEREST STATEMENT

The authors declare that they have no conflicts of interest.

### PEER REVIEW

The peer review history for this article is available at https://www.webofscience.com/api/gateway/wos/peer-review/10.1002/btm2.10515.

## Supporting information


**Data S1:** Supporting Information.

## Data Availability

All data generated or analyzed during this study are included in the published articles.
