# Supplementary material for: Synergistic ferroptosis‐starvation therapy for bladder cancer based on hyaluronic acid modified metal–organic frameworks
Source: Bioeng Transl Med. 2023 Mar 22;8(3):e10515. doi: 10.1002/btm2.10515 (PMC10189452; doi:10.1002/btm2.10515)
Supplement: Supplementary file 1 — Data S1: Supporting Information. [file BTM2-8-e10515-s001.pdf]

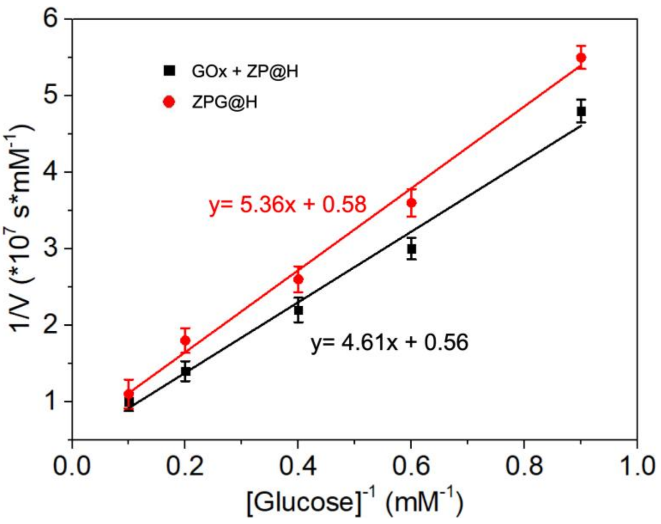

Figure S1. Double reciprocal curves of initial reaction rate with concentrations of glucose, which was catalyzed by GOx + ZP@H or ZPG@H.

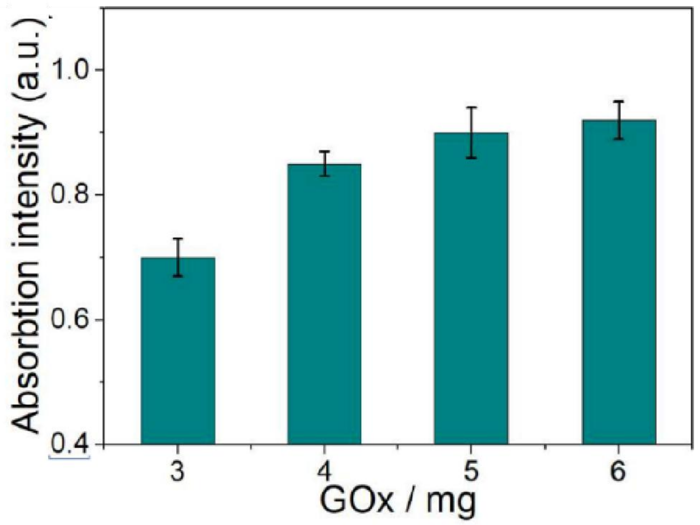

Figure S2. The absorbance intensity at 652 nm of TMB, glucose and ZPG@H with different mass ratios of GOx and PdCuAu.

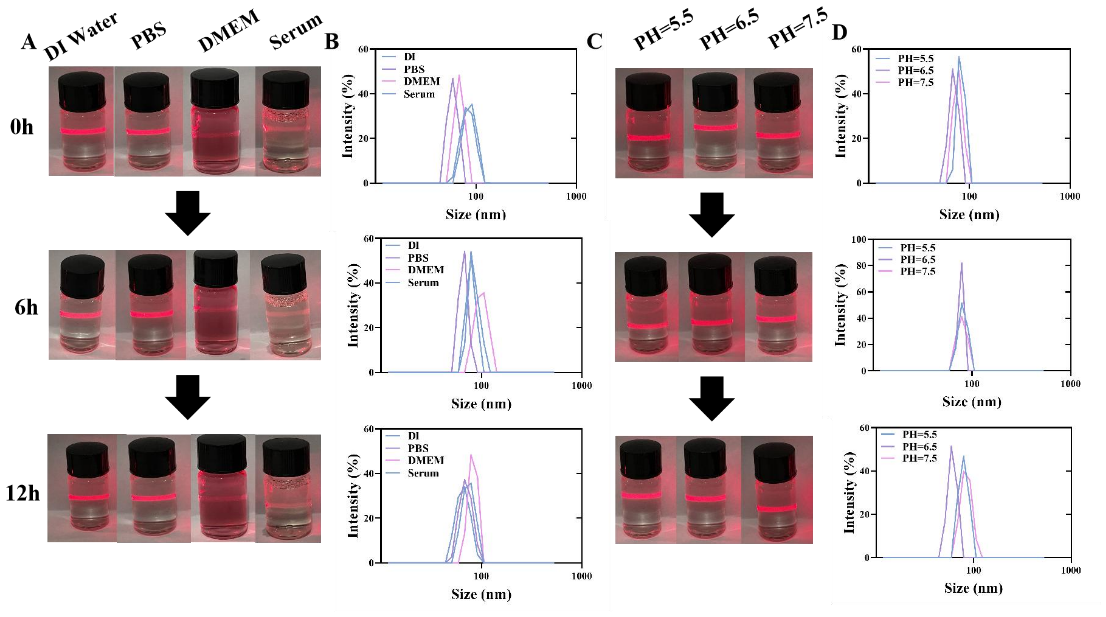

Figure S3. (A). The Tyndall effect of ZPG@H dispersed in DI water, PBS (pH 7.4), DMEM and serum of mice for 0 h, 6 h, 12 h. (B). Hydrodynamic size distribution of ZPG@H after incubation in DI water, PBS (pH 7.4), DMEM and serum of mice for 0 h, 6 h, 12 h. (C). The Tyndall effect of ZPG@H dispersed in PBS (pH 5.5), PBS (pH 6.5), and PBS (pH 7.4)for 0 h, 6 h, 12 h. (D). Hydrodynamic size distribution of ZPG@H after incubation in PBS (pH 5.5), PBS (pH 6.5), and PBS (pH 7.5)for 0 h, 6 h, 12 h.

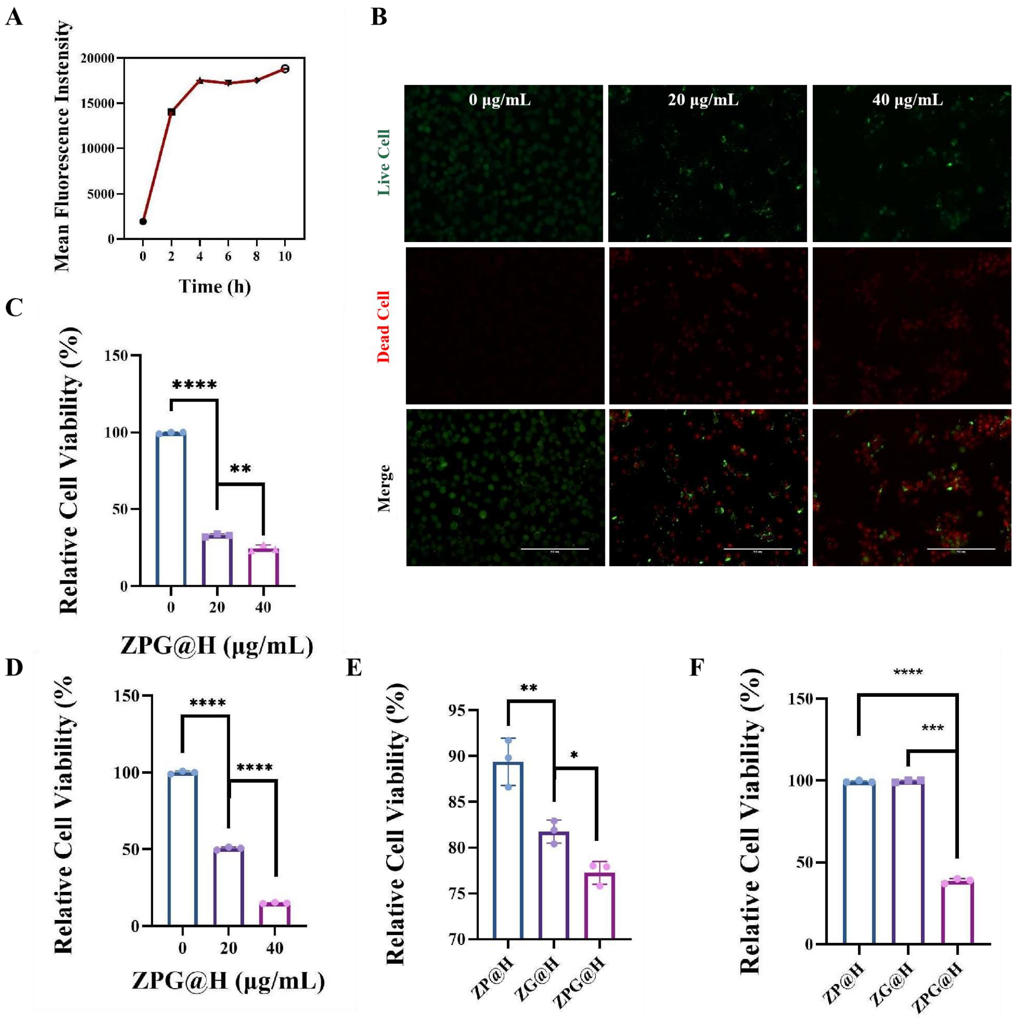

Figure S4. (A) Flow cytometry analysis of RhB-conjugated tumor cells after exposure to ZPG@H (30 μg /mL) for 10 h. (B) Fluorescence imaging of T24 cells treated with different concentrations of ZPG@H for 6h. Live/dead stain showing dead cells as red and live cells as green. The scale bar is 200 μm. (C) Average cell viability of T24 cells treated with different concentrations of ZPG@H for 6h. (D) Flow cytometry analysis of cell viability after treated with different concentrations of ZPG@H for 6h. (E) Flow cytometry analysis of cell viability after treated with ZG@H, ZP@H and ZPG@H (30 μg /mL) for 6h. (F) Average cell viability of T24 cells treated with ZG@H, ZP@H and ZPG@H (30 μg /mL) for 6h. Asterisks indicate significant differences (*p<0. 05, **p<0.01, ***p<0.001).

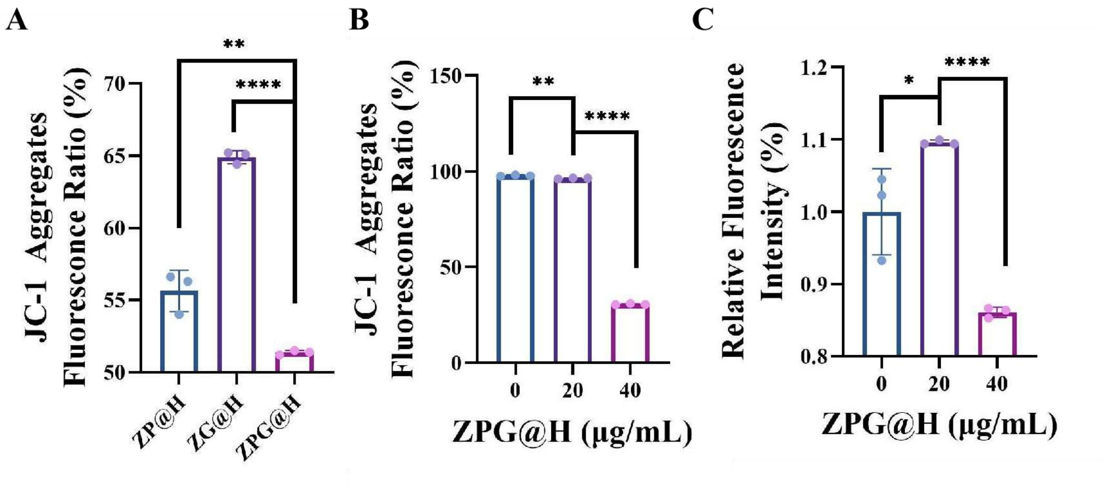

Figure S5. (A) Flow cytometry analysis of JC-1 stained T24 cells treated with ZG@H, ZP@H and ZPG@H (30 μg /mL) for 6h. (B) Flow cytometry analysis of JC-1 stained T24 cells incubated with different concentrations of ZPG@H for 6h. (C) Flow cytometry analysis of ROS probe stained T24 cells incubated with different concentrations of ZPG@H for 6h.

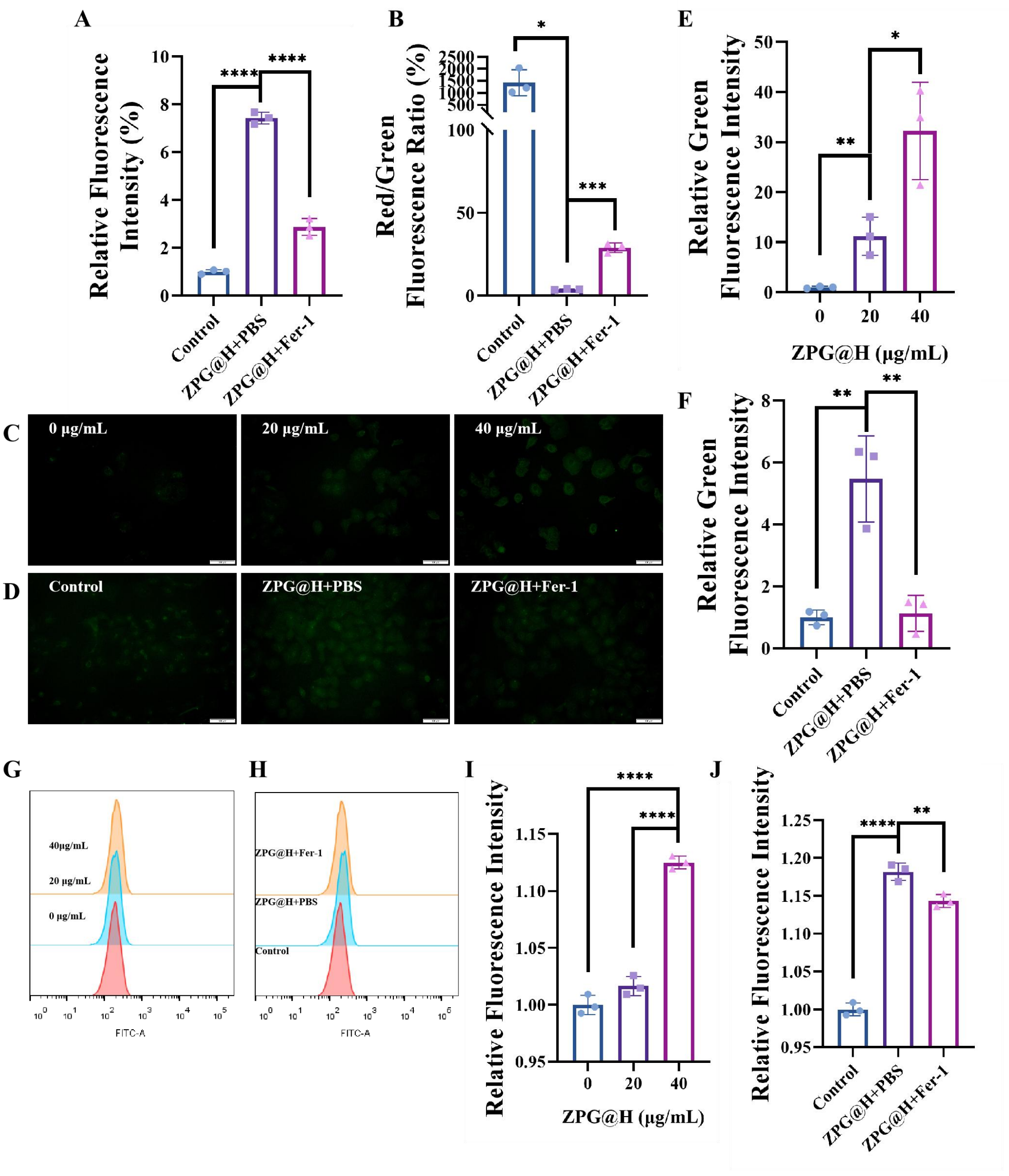

Figure S6. (A) Average red/green fluorescence ratio of JC-1 stained T24 cells treated with ZPG@H (30μg/mL) and Fer-1 (20μM) for 6 h. (B) Average PE-Texas Red fluorescence intensities of ROS probe stained T24 cells incubated with ZPG@H (30μg/mL) and Fer-1 (20μM) for 6h. (C). Fluorescence imaging of Liperfluo stained T24 cells incubated with different concentrations of ZPG@H for 6 h. (D). Fluorescence imaging of Liperfluo stained T24 cells incubated with ZPG@H (30 μg/mL) and Fer-1 (20 μM) for 6 h. (E). Relative green fluorescence intensities of

Liperfluo stained T24 cells incubated with different concentrations of ZPG@H for 6 h. Asterisks indicate significant differences (*p < 0. 05, **p < 0.01, ***p < 0.001). (F). Relative green fluorescence of Liperfluo stained T24 cells treated with ZPG@H (30 μg/mL) and Fer-1 (20 μM) for 6 h. Asterisks indicate significant differences (*p < 0. 05, **p < 0.01, ***p < 0.001). (G). Flow cytometry analysis of Liperfluo stained T24 cells treated with different concentrations of ZPG@H for 6 h. (H). Flow cytometry analysis of Liperfluo stained T24 cells treated with ZPG@H (30 μg/mL) and Fer-1 (20 μM) for 6 h. (I). Average green fluorescence of Liperfluo stained T24 cells incubated with different concentrations of ZPG@H for 6 h. (J). Average green fluorescence of Liperfluo stained T24 cells incubated with ZPG@H (30 μg/mL) and Fer-1 (20 μM) for 6 h.

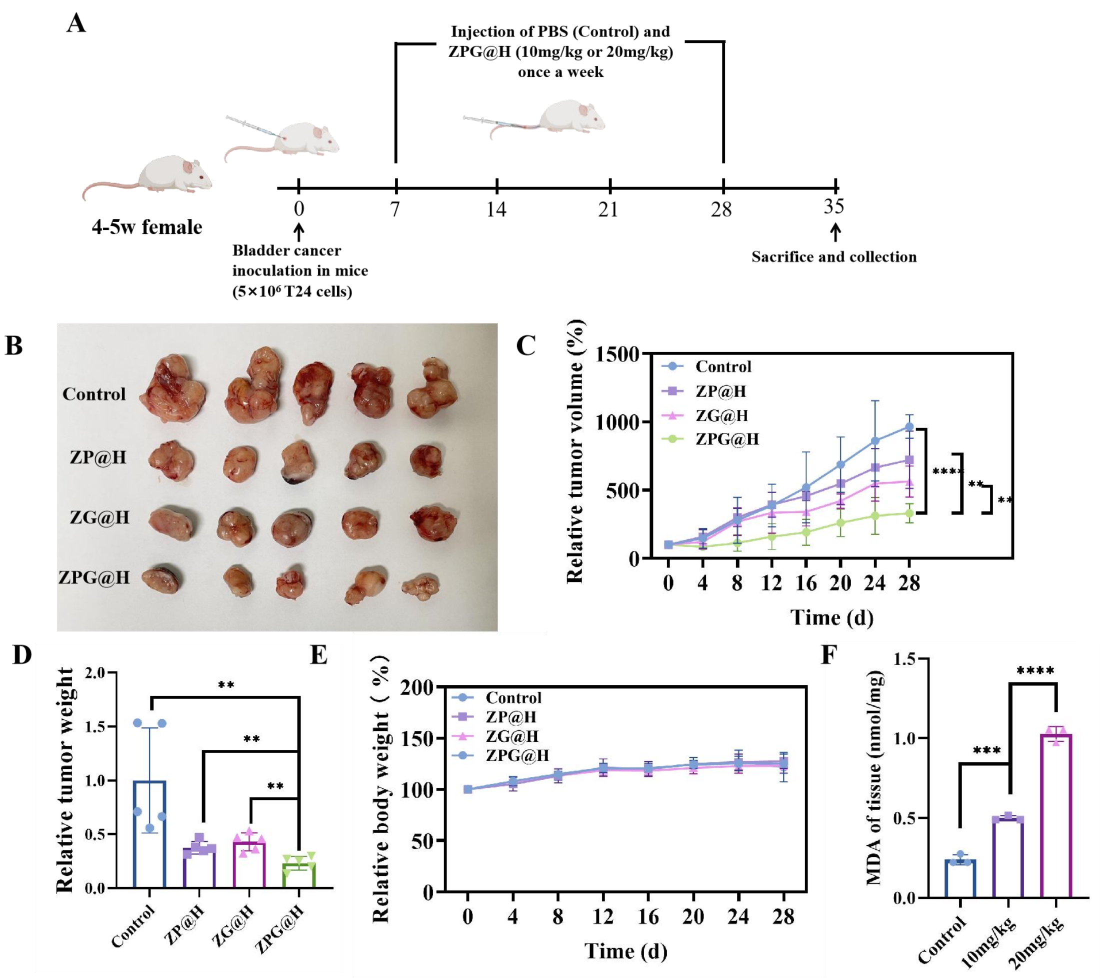

Figure S7. (A) Schematic diagram of the mouse model. (B) Images of the primary tumors derived from mice that underwent different treatments (1: PBS; 2: ZP@H; 3: ZG@H; 4: ZPG@H) (n = 5). (C)The detection of tumor volume after the indicated treatments (n = 5). (D)The detection of tumor weight after the indicated treatments (n = 5). (E). The detection of body weight of mice after the indicated treatments (n = 5). (F). MDA content in tumor issues following the treatment with different concentration of ZPG@H. Asterisks indicate significant differences (*p < 0. 05, **p < 0.01, ***p < 0.001).

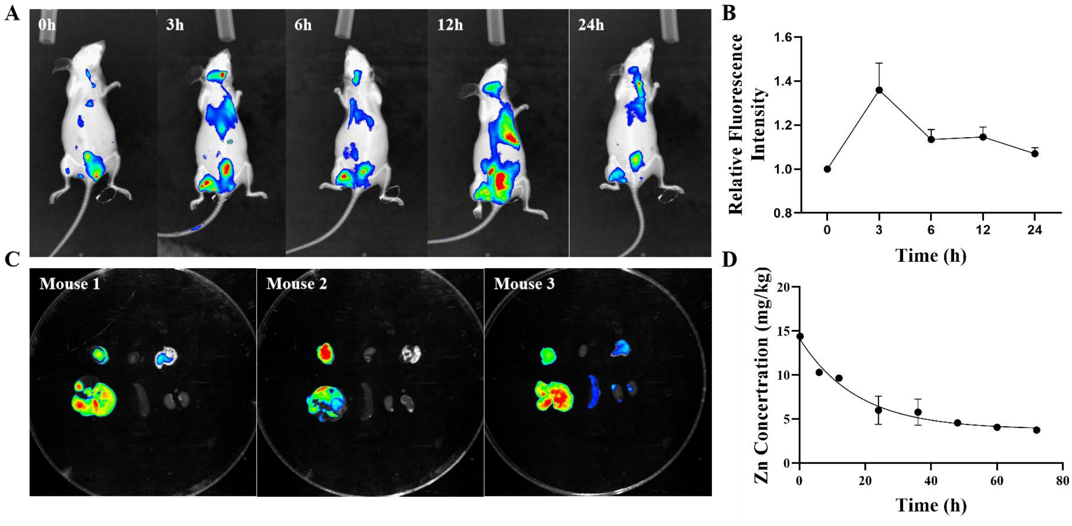

Figure S8. (A). Fluorescence images of T24-tumor-bearing mice at 0, 3, 6, 12, and 24 h after treatment with ZPG@H (n = 3). (B) Average fluorescence intensity of T24-tumor-bearing mice at 0, 3, 6, 12, and 24 h after treatment with ZPG@H (n = 3). (C). Fluorescence images of major organs from the mice 6 h post-injection (n = 3). (D). Time-concentration curve of zinc in blood from mice after a single dose iv administration of 10 mg kg ZPG@H (n = 3).

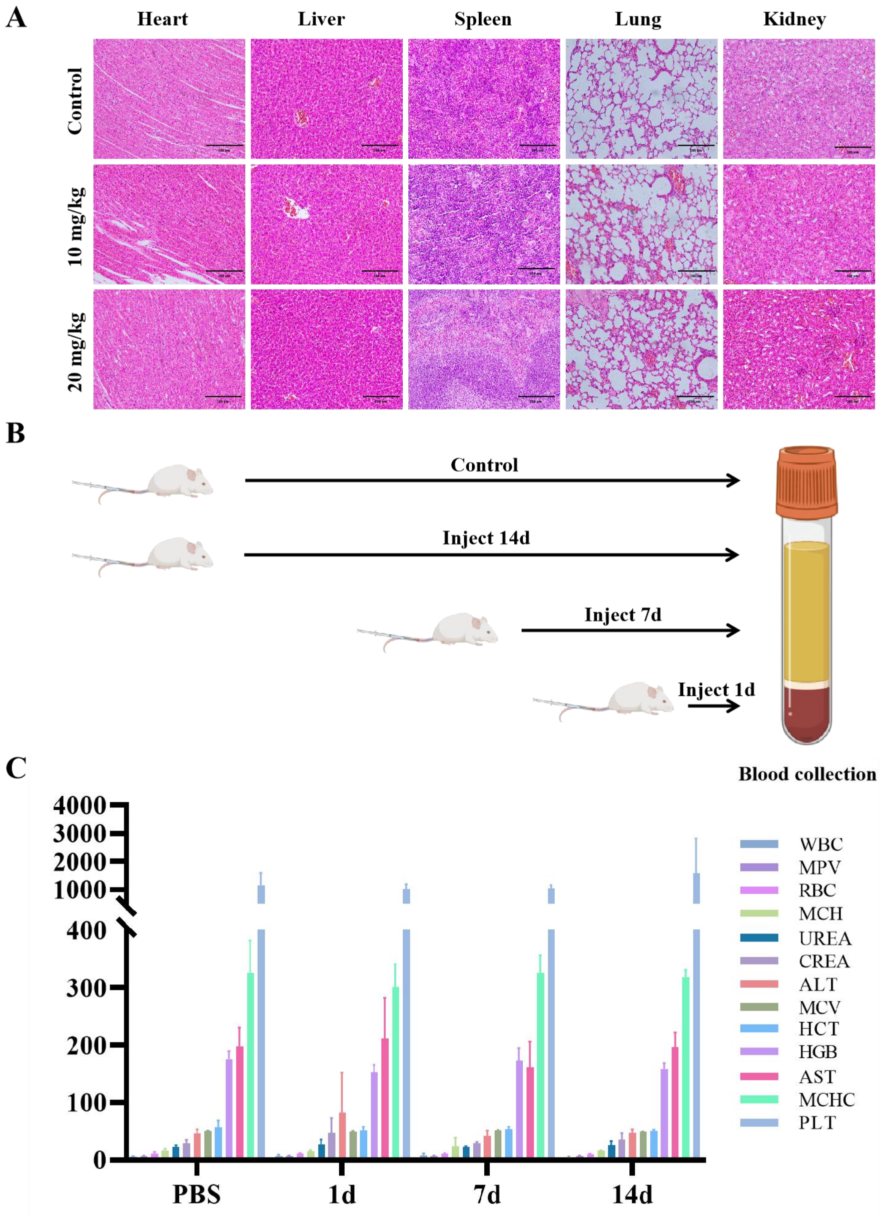

Figure S9. (A) H&E staining of the main organs of mice incubated with tumors after different interventions. The scale bar represents 200 μm. (B) Time axis of ZPG@H injection through tail vein in mice. (C) Routine blood and blood biochemical analysis of the healthy mice intravenously injected with ZPG@H (20 mg/kg) at 1, 7 and 14 days post-injection (The units of ALT, AST are U/L; the units of CREA is μmol/L; the units of BUN, WBC, RBC, HGB, HCT, MCV, MCH, MCHC, and PLT are mg/dl, 10^9/L, 10^{12}/L, g/L, %, fL, pg, g/L, and 10^9/L, respectively.).
